# Phylogenetic and Structural Analysis of Porcine Circovirus Type 2 from 2016 to 2021 in Jilin Province, China

**DOI:** 10.3390/microorganisms11040983

**Published:** 2023-04-10

**Authors:** Si Chen, Xue Li, Liying Zhang, Jiawei Zheng, Lin Yang, Guyu Niu, Huimin Zhang, Ying Ren, Jing Qian, Changjiang Sun, Linzhu Ren

**Affiliations:** 1 College of Animal Sciences, Key Laboratory for Zoonoses Research, Ministry of Education, Jilin University, 5333 Xi’an Road, Changchun 130062, China; 2 Public Computer Education and Research Center, Jilin University, 5333 Xi’an Road, Changchun 130062, China; 3 Institute of Veterinary Medicine, Jiangsu Academy of Agricultural Sciences, Nanjing 210014, China; 4 College of Veterinary Medicine, Jilin University, 5333 Xi’an Road, Changchun 130062, China

**Keywords:** porcine circovirus type 2 (PCV2), molecular epidemiology, epitope, mutation

## Abstract

Porcine circovirus disease (PCVD) caused by porcine circovirus type 2 (PCV2) is widely distributed in pig farms. Up until now, nine genotypes of PCV2, PCV2a to 2i, have been identified in diseased pigs worldwide. This study analyzed 302 samples collected in the Jilin Province of China from 2016 to 2021, followed by genetic analysis of the PCV2 isolates. Meanwhile, the antigen epitopes, amino acid mutations, 3D structure of the PCV2 isolates and commercially available vaccine strains were evaluated and compared. The results showed that the predominant genotypes of PCV2 were PCV2b, followed by PCV2e and PCV2d in Jilin Province during 2016–2021. Although mutations were detected in the isolates, no recombination occurred in the PCV2 isolates, indicating a stable genotype of PCV2 in Jilin Province during these years. Moreover, the B cell epitopes in the Cap and Rep proteins of eighteen PCV2 isolates and T cell epitopes in the Cap of the isolates were changed compared to three currently used vaccine strains. The mutations in the Cap and Rep proteins did not affect their spatial conformation. Therefore, bivalent or multivalent vaccines with different genotypes of PCV2 might improve the protective effect of vaccines.

## 1. Introduction

Porcine circovirus disease (PCVD) is an infectious disease of pigs caused by porcine circoviruses (PCVs), which occur in pig farms worldwide [1,2]. PCVs are single-strand circular DNA viruses belonging to the *Circovirus* genus *Circoviridae* family, which contains four genotypes, namely PCV1, PCV2, PCV3, and PCV4 [1,2,3]. Among the PCVs, PCV2 is the most widely distributed and has caused severe disease and economic losses worldwide. The clinical symptoms of pigs infected with PCV2 are immunosuppression and multiple diseases, including porcine dermatitis and nephropathy syndrome (PDNS), postweaning multisystemic wasting syndrome (PMWS), and porcine respiratory disease complex (PRDC) [2]. PCV2 was first identified in pigs associated with wasting syndromes in North America and Europe in 1998, which was later genotyped as PCV2a and gradually caused epidemics worldwide [4]. Since 2003, another genotype of PCV2, PCV2b, has been discovered and has quickly become a prevalent genotype of PCV2 among pigs. PCV2c was found in the serum of healthy pigs in Denmark, followed by Brazil, China, and the Netherlands [5,6,7,8]. After that, another six genotypes, including PCV2d, 2e, 2f, 2g, 2h, and 2i, were identified in both sick and healthy pigs in recent years [9,10]. To date, considerable evidence shows that PCV2d has gradually replaced PCV2b as the predominant genotype globally or circulates as a co-infection with PCV2b in some areas or countries [10,11,12]. Furthermore, although there was no significant difference in the level of PCV2 viremia and the occurrence of PCV2-related lymphopathy in pigs inoculated with PCV2a, PCV2b, and PCV2d, the co-infection with other pathogens, such as *Mycoplasma hyopneumoniae* and porcine reproductive and respiratory syndrome virus, might enhance the PCV2-related diseases [13,14]. Therefore, it is necessary to continuously monitor and genotype PCV2 to evaluate the epidemic and adopt an appropriate strategy to prevent and control the disease.

Vaccination is the most effective way to prevent and control epidemic diseases. The currently available PCV2 vaccines are mainly based on PCV2a or PCV2b [10,15]. Although several groups reported that cross-protection between heterologous genotypes exists and that PCV2a or PCV2b-based vaccines could protect pigs against PCV2a, 2b, or even 2d infection [16,17,18], previous researches are still controversial [10,19,20]. For example, the effectiveness of the PCV2 vaccine in protecting animals from the infection of the heterologous genotype or mutated strain declined compared with the homologous genotype [10,19,20]. The possible reason is that the critical amino acid mutation of antigen epitope or the difference of epitopes between genotypes leads to decreased vaccine protection efficiency. Therefore, comparing the differences in antigen epitopes and protein structures between vaccine and epidemic strains is also helpful in designing and preparing high-efficiency vaccines.

The genome of PCV2 contains 11 open reading frames (ORFs), of which the Rep and Cap proteins, encoded by the ORF1 and ORF2, are the main proteins [21]. Rep is a replicase, and Cap is the only structural protein that contains the majority of the antigenic epitopes and is responsible for immune stimulation [1,22]. This study analyzed the clinical samples collected in the Jilin Province of China from 2016 to 2021, followed by the genotyping and genetic diversity analysis of the PCV2 isolates. The antigen epitopes, amino acid mutations, 3D structure of the PCV2 isolates, and commercially available vaccine strains were also predicated and compared. This study provides a reference for evaluating the genetic evolution of PCV2 and the selection and development of PCV2 vaccines in Northeast China.

## 2. Materials and methods

### 2.1. Sample Collection, Viral DNA Extraction, and PCV2 Detection

Three hundred and two samples, including two hundred and eighteen samples (lymph node or tonsil) from sick pigs and eighty-four serum samples from healthy pigs, were randomly collected from twenty-one pig farms between 2016 and 2021 in Jilin province, China. Pigs with obvious clinical symptoms were identified as sick pigs, and those without clinical signs were treated as healthy. Tissue samples (lymph node or tonsil) from nine to twelve sick animals and serum samples from four healthy animals were collected from each pig farm. Total viral DNA was extracted directly from tissue samples using a TIANamp Genomic DNA Kit (Tiangen, Beijing, DP304). All samples were screened for PCV2 with PCR using the primers F1/R1 (F1: 5′-GGGCCAGAATTCAACCTTAACC-3′, R1: 5′-CGCACCTTCGGATATACTGTCA-3′, GB/T 34745-2017). PCR was performed with 10 μL 2 × PCR MasterMix (Tiangen, Beijing, China), 2 μL primers F1/R1 (10 μmol/L each), 1 μL DNA, and 7 μL ddH_2_O. The PCR was conducted as follows: predenaturation at 94 °C for 3 min; 94 °C 30 s, 57 °C 30 s, 72 °C 12 s, 35 cycles; elongation at 72 °C for 5 min. The PCR product was evaluated via 0.8% agarose gel electrophoresis.

Moreover, eighteen isolates were randomly selected for full-length genome amplification using the primer F2/R2 (F2′: 5-ACCAGCGCACTTCGGC-3′, R2: 5-AATACTTACAGCGCACTTCTTTCG-3′), followed by cloning into the pLB plasmid (Tiangen, Beijing, VT205) and Sanger sequencing. Sanger sequencing was performed using the Applied Biosystems 3730xl by Comate Bioscience Co., Ltd. (Changchun, China). The primers F2/R2 were designed in this study according to the sequence of PCV2 CC1 (GenBank ID: JQ955679.1) and synthesized by Comate Bioscience Co., Ltd. (Changchun, China). PCR was performed with 10 μL 2 × PCR MasterMix (Tiangen, Beijing, China), 2 μL primers F1/R1 (10 μmol/L each), 1 μL DNA, and 7 μL ddH_2_O. The PCR was conducted as follows: predenaturation at 98 °C for 30 s; 98 °C 10 s, 52 °C 30 s, 72 °C 40 s, 35 cycles; elongation at 72 °C for 5 min. The complete genome sequences of the 18 PCV2 strains used in this study have been deposited in GenBank: ON012605-ON012608, OP233007-OP233015, OP279264-OP279268 (Supplementary Table S1).

### 2.2. Sequence Alignments and Phylogenetic Analyses

For the full-length genome alignment, thirty representative strains of different genotypes of PCV2 were collected (Supplementary Table S2), and eighteen PCV2 isolates were analyzed. A maximum likelihood (ML) tree was reconstructed using MEGA X with the TN93 + G + I model and 1000 bootstrap replicates [23]. Nucleotide acid sequences of the PCV2 ORF1 and ORF2 were analyzed using MEGA X. An ML tree was reconstructed with the indicated best-fitting model and 1000 bootstrap replicates. The model with the lowest Bayesian information criterion (BIC) value was identified as the best-fitting. Therefore, the TN93 + G + I model was used for the full-length genome; the K2 + G + I model and HKY+G model were used for the ORF1 and ORF2, respectively.

### 2.3. Recombination Analyses

Recombination analysis was performed with the full-length genomic sequences of 18 PCV2 isolates and reference sequences (Supplementary Table S2) using RDP v4.101 and Simplot v.3.5.1. A total of seven methods, including RDP [24], GENECONV [25], Bootscan [26], Maxchi [27], Chimaera [28], SiSscan [29], and 3Seq [30], were implemented to detect the recombination events in RDP V4.101. The *p*-value was set to 0.05. Recombination had to be confirmed with a strict standard by at least five of the seven methods. Simplot v.3.5.1 was conducted as follows: Window size 200 bp, step size 20 bp, replicates 1000 times, and distance model Kimura (2-parameter). Notably, reference strain DQ233257 may be a recombinant, and the sequence was removed from the recombination analysis.

### 2.4. Epitope Prediction and Mutation Analysis

A total of eighteen PCV2 sequences were aligned with three commercially inactivated PCV2 vaccine strains SH (AY686763, PCV2b), DBN-SX07 (HM641752, PCV2b), and LG (HM038034, PCV2a) in China. 

Epitope prediction was performed to evaluate the B cell epitopes of the Cap and Rep proteins from different circoviruses using the online server ABCpred (https://webs.iiitd.edu.in/raghava/abcpred/ABC_submission.html accessed on 10 September 2022) [31,32]. The threshold was set to 0.7. The length of the B cell epitopes was set to 10 amino acids (aa). The T cell epitopes of the Cap and Rep proteins were predicted using the online server NetCTL-1.2 (https://services.healthtech.dtu.dk/service.php?NetCTL-1.2, accessed on 10 September 2022). 

A PCV2 Cap and Rep mutation analysis was performed on the predicted structures using Missence3D (http://missense3d.bc.ic.ac.uk/~missense3d/, accessed on 5 October 2022).

### 2.5. Three-Dimensional (3D) Structures Modeling

The domains of the Cap and Rep proteins of the 18 PCV2 isolates and three reference strains, AY391729 (PCV2b), AY291317 (PCV2d), and GU001709 (PCV2e), were predicted using Pfam (http://pfam.xfam.org/, accessed on 30 August 2022). The mutations in the Cap and Rep proteins of eighteen isolates (nine PCV2b, three PCV2d, and six PCV2e) were also compared with three early reported PCV2 strains (PCV2b AY391729, PCV2d AY291317, and PCV2e GU001709) of each genotype in China. Three-dimensional (3D) structures of domains of the Cap and Rep proteins were modeled using the online software I-TASSER (https://seq2fun.dcmb.med.umich.edu/I-TASSER/, accessed on 30 August 2022) [33,34]. The 3D structural alignment was performed using the online program Pymol 2.0. In addition, an RMSD (Schrödinger, New York, NY, USA) analysis for the structural alignment of the viral proteins was conducted according to the protocol described by Souza et al. [31,32].

## 3. Results

### 3.1. Dominant Genotypes of PCV2 in Jilin Province during 2016–2021

As a result, PCV2 was detected in 218 tissue samples and 42 blood samples, and the detection rate of PCV2 was 86.09% (260/302). These results indicated that the infection rate of PCV2 in pigs in Jilin Province was high.

**Figure 1:**
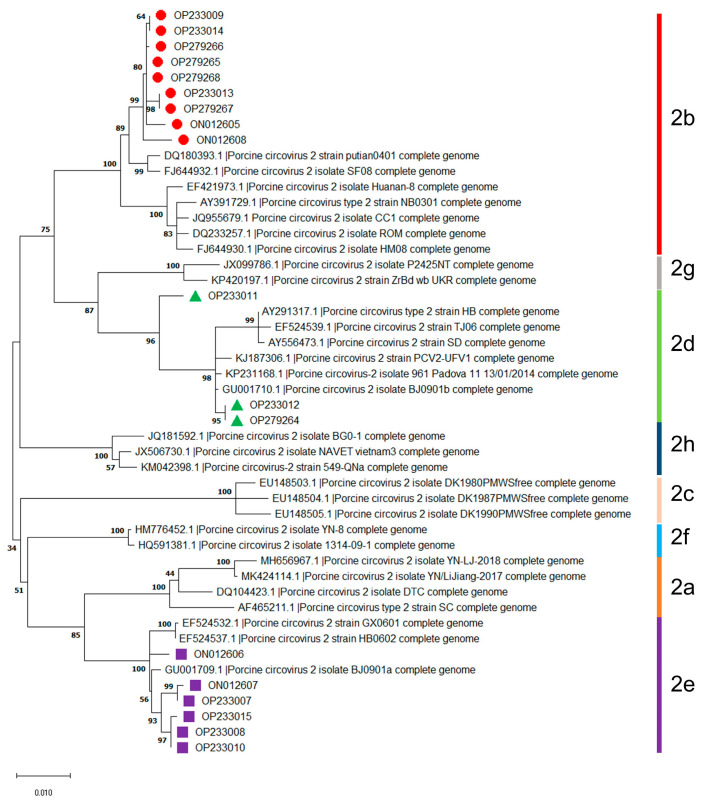
Genotyping of PCV2 isolates obtained during 2016–2021 in Jilin province, China. Information on the thirty available reference sequences of the PCV2 genotypes are summarized in Supplementary Table S2.

The randomly selected eighteen isolates can be divided into three genotypes, including PCV2b (9 isolates), PCV2d (3 isolates), and PCV2e (6 isolates), based on thirty available reference sequences of PCV2 genotypes (Figure 1). These results indicated that the dominant genotypes of PCV2 were PCV2b, followed by PCV2e and PCV2d in Jilin Province during 2016–2021.

### 3.2. Recombination Analysis

**Figure 2:**
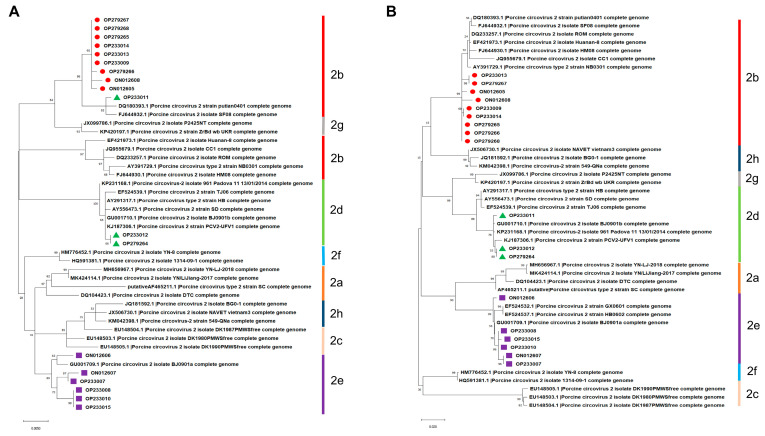
Phylogenetic trees constructed based on the nucleotide sequences of PCV2 ORF1 and ORF2. (**A**) ORF1. (**B**) ORF2.

As shown in Figure 2A, isolate OP233011 in the phylogenetic tree based on the nucleotide sequences of the *ORF1* gene was grouped into PCV2b but not in PCV2d, which was different from the above result in Figure 1. On the contrary, phylogenetic trees based on the nucleotide sequences of ORF2 were similar to the phylogenetic trees based on the full-length genome of PCV2 mentioned above in Figure 1 (Figure 2B). These results indicate that the nucleotide sequences of the full-length genome and ORF2 but not the ORF1 of PCV2 are more suitable for the genotyping and phylogenetic analysis of PCV2. 

**Figure 3:**
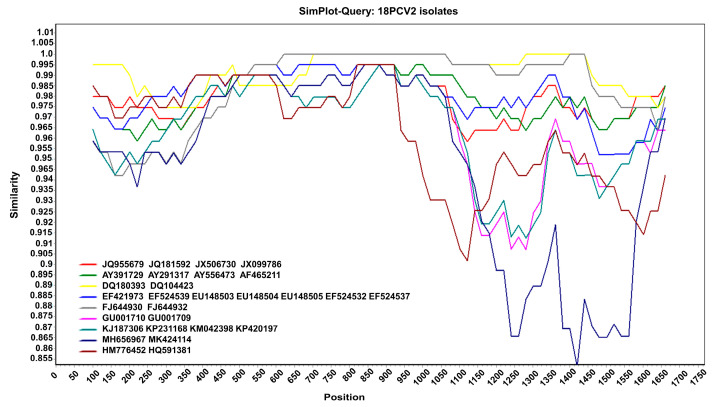
Recombination analysis. Nucleotide sequence similarity was assessed using SimPlot v.3.5.1. Sequence similarity between the 18 PCV2 isolates and 29 reference sequences.

Moreover, the results of the recombination analysis showed that no consistent recombination event was detected among the 18 isolates via the seven methods (Table 1). This result was further confirmed using SimPlot v.3.5.1. As shown in Figure 3, there was no crossover point or recombination between the 18 PCV2 isolates and reference sequences. Therefore, no recombination occurred in the PCV2 isolated in the Jilin Province from 2016 to 2021. The genotype of PCV2 was relatively stable during these years.

### 3.3. Epitopes Predication

**Figure 4:**
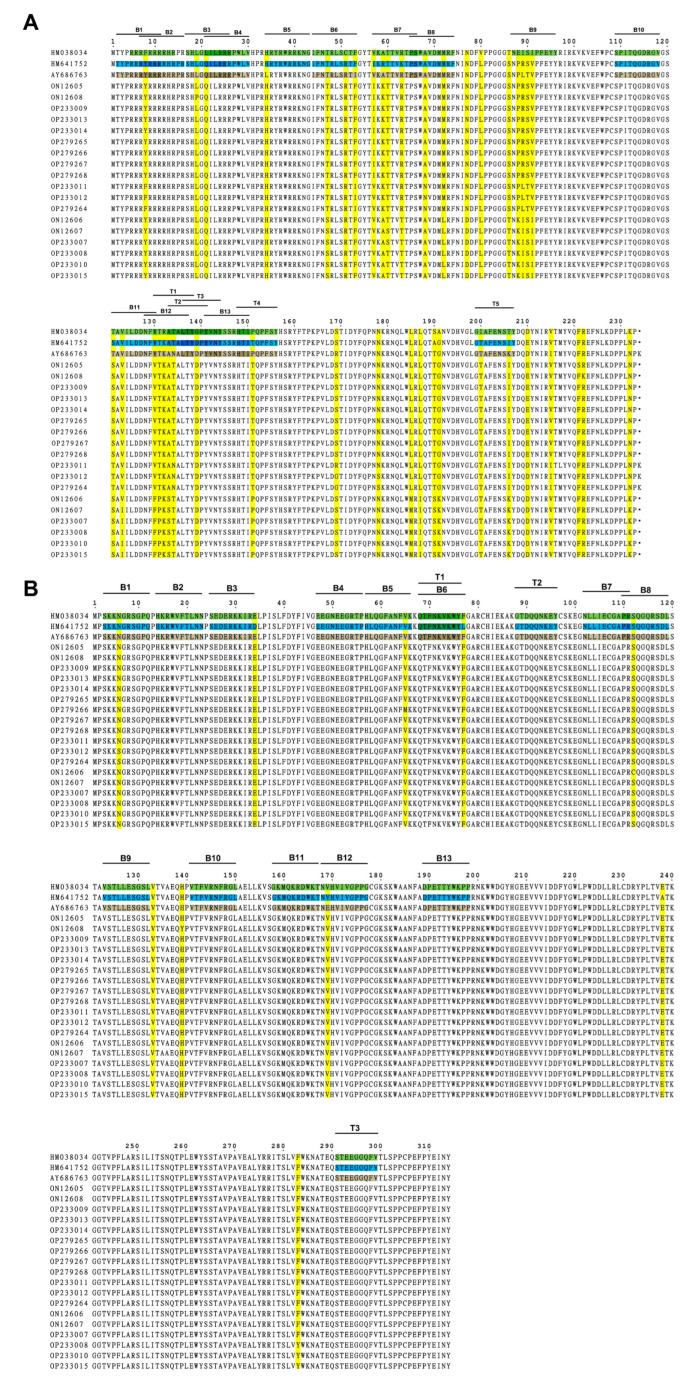
Mutation and epitope analysis of the PCV2 isolates. (**A**) Amino acid sequence alignment and potential epitopes of the PCV2 Cap protein. (**B**) Amino acid sequence alignment and potential epitopes of the PCV2 Rep protein. The epitopes of AY686763 Cap/Rep protein are brown, and that of HM641752 and HM038034 Cap/Rep protein are labeled in blue and green, respectively. Mutations are highlighted in yellow. B, B cell epitope; T, T cell epitope.

Amino acid sequences of viral Cap and Rep proteins of eighteen PCV2 isolates were aligned with three currently used PCV2 commercial inactivated vaccine strains, SH (AY686763, PCV2b), DBN-SX07 (HM641752, PCV2b), and LG (HM038034, PCV2a), in China, respectively, followed by epitope analysis via the online software ABCpred and NetCTL. For the Cap protein (Supplementary Tables S3–S5, Figure 4A), the mutation mainly occurred at the residues Y8F, L19P, L21Q, T47S, I53F, V57I, A68N, T121S, and V123I of the eighteen PCV2 isolates compared with that of the inactivated vaccine strain SH. Compared with the inactivated vaccine strain DBN-SX07, the mutation sites were located at the residues Y8F, L19P, T47S, I57V, A68N, S121T, V123I, and V130F of the PCV2 isolates. The mutation sites were F8Y, L19P, L21Q, T47S, V57I, A68N, E88P, E88K, I89L, I89R, S90T, and I91V of the PCV2 isolates compared to the inactivated vaccine strain LG. 

For the Rep protein (Supplementary Tables S3–S5, Figure 4B), fewer mutations were detected in the Rep protein than in the Cap protein. The residues of N65S, V65M, S112C, and E169V were identified in the eighteen PCV2 isolates compared with that of the inactivated vaccine strain SH. Compared with the inactivated vaccine strain DBN-SX07, the mutations were at N65S, D34E, V65M, and S112C in the eighteen PCV2 isolates. The mutations mainly occurred at N65S, V65M, and S112C in the PCV2 isolates compared with the inactivated vaccine strain LG. 

Furthermore, thirteen B and five T cell epitopes were identified in the Cap protein (Supplementary Tables S3–S5, Figure 4). Thirteen B and three T cell epitopes were detected in the Rep protein. No mutation in the T cell epitope of the Rep protein was observed. Moreover, the B cell epitopes B7 (V^57^KATTVRTPS^66^), B9 (N^87^EISIPFEYY96), and B12 (N^128^FVTRATALT^137^) of the Cap proteins contain more mutations. These results demonstrated that the compositions of B cell epitopes in the Cap and Rep proteins of the eighteen PCV2 isolates and T cell epitopes in the Cap of PCV2 were changed compared to the three vaccine strains. Therefore, the mutations in the Cap proteins of the isolates may affect the epitopes of the Cap proteins, resulting in the shielding of the neutralizing antibodies’ epitopes and decreasing vaccine protection. 

It is worth noting that more mutations were found in the eighteen isolates (PCV2b, PCV2d, and PCV2e) and two PCV2b vaccine strains compared with the PCV2a vaccine strain LG (PCV2a), which may weaken the protective effect of the PCV2a-based vaccine against PCV2b infection. However, these results still need to be verified using in vivo experiments.

### 3.4. Mutations Analysis of Cap and Rep Proteins 

The mutations in the Cap and Rep proteins of the eighteen isolates were also compared with three early reported PCV2 strains (AY391729, AY291317, and GU001709) of each genotype in China (Supplementary Table S2), followed by the spatial conformation analysis of the proteins. As shown in Table 2, compared with the reference PCV2b strain AY391729, three mutations in the Cap protein, R59K, K63R, and K180R, were the consensus among nine PCV2b isolates. In contrast, an additional differential residue, F221S or L19P, was found in the isolates ON012605 and ON012608, respectively. 

Compared with the reference PCV2d strain AY291317, three common mutations, including Y8F, A59K, and P151T, were identified in the Cap of the PCV2d isolates OP233011, OP233012, and OP279264. Mutation M217L in the PCV2d OP233011 and R169G and N178S of the PCV2d strains OP233012 and OP279264 contributed to the genetic distance between the three isolates. 

For six PCV2e isolates, the V123I mutation was detected in five isolates, including OP233015, OP233008, OP233010, OP233007, and ON012607, compared with the reference strain PCV2e GU001709. An additional mutation, T60S, occurred in the isolates OP233007 and ON012607. However, the amino acids of the Cap proteins were identical between isolate ON012606 and the reference strain PCV2e GU001709. 

In addition, the common mutations in the Rep protein were D34E and L77F between nine PCV2b isolates and the reference strain PCV2b AY391729. The residues of the Rep protein were the same between the PCV2d and PCV2e isolates and the reference strains. These results further confirmed that the PCV2 Rep is more conserved than the viral Cap.

To analyze whether the PCV2 Cap and Rep protein mutations affect their structure and function, we modeled the domains of the Cap and Rep protein of PCV2 (Supplementary Figure S1). For the Cap protein, the RMSD values of AY391729 and ON012605 were 0.427 Å. The RMSD values of AY391729 and ON012608 were 0.516 Å. Meanwhile, the RMSD values of AY391729 and ON012608 were 0.453 Å. The RMSD values of AY291317 with OP233011 and OP233012 were 0.429 Å. The RMSD values of GU001709 and OP233015 were 0.361 Å. Meanwhile, the RMSD values of GU001709 and OP233007 were 0.385 Å.

For the Rep protein, the RMSD values of AY391729 and OP279266 were 0.230 Å. the RMSD values of AY391729 and OP279268 were 0.266 Å. These results indicated that the mutations in the Cap and Rep proteins might not affect the spatial conformation of the proteins.

Moreover, the secondary structures of the viral Cap and Rep proteins of the eighteen isolates and the corresponding reference strains were further analyzed using Missence3D (Table 3). The results showed that the substitution in the isolates did not alter the secondary structure and conformations of the ‘E’ (extended strand in parallel and/or anti-parallel β-sheet conformation), ‘T’ (hydrogen bonded turn), ‘G’ (3-turn helix), or ‘H’ (4-turn helix), compared to the reference strains. However, the viral proteins of each isolate and the reference strains showed different degrees of difference in some aspects, such as crash, buried hydrophilic introduced, buried charge introduced, buried charge switch, disallowed phi/psi, buried charge replaced, buried H–bond breakage, cavity altered, and buried/exposed switch, etc. These results indicate that the mutations in the Cap and Rep proteins of the eighteen isolates did not destroy their structure and conformation. Still, the binding activities of viral proteins with target proteins might differ.

## 4. Discussion

This study detected and sequenced PCV2 in the diseased pigs collected recently in Jilin Province and its surrounding areas. As a result, PCV2 was detected in 218 tissue samples and 42 blood samples, and the detection rate of PCV2 was 86.09% (260/302), suggesting a high infection rate in Jilin Province. However, we found that the genotyping of PCV2 based on the full-length sequence and ORF2 differed from the ORF1. Nucleotide sequences of the full-length genome and the ORF2 of PCV2 are more suitable for the genotyping and phylogenetic analysis of the virus. Further study showed that the dominant genotypes of PCV2 were PCV2b, followed by PCV2e and PCV2d in Jilin Province during 2016–2021. 

Recombination is an essential evolutionary path of the virus’s infection cycle, which enables the virus to escape from the host’s immune system and survive. Since 2007, several recombination events of PCV2 have been reported [35,36,37], which occurred within and between genotypes. Furthermore, PCV2 is a DNA virus with a high genetic evolution rate. The evolutionary rate of the PCV2 *Cap* gene has been estimated to be 1.102–1.2 × 10^−3^ substitutions/site/year [38,39]. The higher evolution rate of PCV2 makes it easier to recombine and generate new genotypes. It was reported that about 33% of PCV2 circulating strains all around the globe are recombinants [40]. However, we found no recombination in the PCV2 isolated in the Jilin Province from 2016 to 2021. These results suggest that the genotype of PCV2 was relatively stable during these years.

Meanwhile, the mutation of the virus’s structural protein may lead to immune escape or declined vaccine protection. Therefore, the amino acid sequences of viral Cap and Rep proteins of eighteen PCV2 isolates were aligned with three currently used PCV2 commercial inactivated vaccine strains, SH (AY686763, PCV2b), DBN-SX07 (HM641752, PCV2b), and LG (HM038034, PCV2a), respectively, followed by epitope analysis. As expected, different mutations were detected in the isolates, including several consensus mutations, compared with the inactivated vaccine strains. It is worth noting that more mutations were found in the eighteen isolates (PCV2b, PCV2d, and PCV2e) and two PCV2b vaccine strains compared with the PCV2a vaccine strain LG (PCV2a), which may weaken the protective effect of the PCV2a-based vaccine against PCV2b infection. However, these results still need to be verified using in vivo experiments.

Currently, PCV2 vaccines mainly include three types: virus-inactivated, subunit, and chimeric. Most commercial vaccines belong to the PCV2a or 2b genotype, including the ZJ/C strain, SH strain, LG strain, WH strain, DBN-SX07 strain, and 1010 strain. It was reported that PCV2a or PCV2b-based vaccines could protect pigs against PCV2a, 2b, or even 2d infection [16,17,18]. We previously found that antisera prepared from the recombinant Caps of PCV2, PCV3, and PCV4 cross-reacted with different PCVs [41]. Furthermore, although PCV2 vaccines based on a single genotype may offer cross-protection, they may not provide enough coverage for the evolving field viruses [19,42]. On the contrary, the bivalent PCV2 vaccine may provide the best opportunity for expanding the coverage to circulating strains of PCV2 [19,43]. Notably, thirteen B cell epitopes and five T cell epitopes were identified in the Cap protein, and thirteen B and three T cell epitopes were detected in the Rep protein. Consensus or overlapped epitopes exist in different PCV2 genotypes. The compositions of the B cell epitopes in the Cap and Rep proteins of the eighteen PCV2 isolates and T cell epitopes in the Cap of the isolates were changed compared to three currently used vaccine strains. However, the mutations in the Cap and Rep proteins did not affect their spatial conformation. In addition, the critical residues that might affect its function must be evaluated. Therefore, whether the mutations in the B and T cell epitopes are the hot spots of mutation and whether this impacts vaccine protection needs further verification. Noteworthy, since PCV2d, 2b, and 2a are the top three genotypes prevalent in pigs worldwide, and PCV2a spreads in wild boars, the spillover and recombination of different PCV2 genotypes may occur between wild boars and pigs [2,44,45]. Therefore, bivalent or multivalent vaccines with diverse genotypes of PCV2 might improve the protective effect of vaccines. It is necessary to monitor the genotype and genetic variation of PCV2 to provide more effective prevention and control of the disease. 

## 5. Conclusions

In conclusion, this study showed that the predominant genotypes of PCV2 were PCV2b, followed by PCV2e and PCV2d in Jilin Province during 2016–2021. Although mutations were detected in the isolates, no recombination occurred in the PCV2 isolated in the Jilin Province from 2016 to 2021, suggesting a stable genotype of PCV2 during these years. Moreover, the B cell epitopes in the Cap and Rep proteins of the eighteen PCV2 isolates and T cell epitopes in the Cap of the isolates were changed compared to three currently used vaccine strains. The mutations in the Cap and Rep proteins did not affect their spatial conformation. Therefore, bivalent or multivalent vaccines with different genotypes of PCV2 might improve the protective effect of vaccines.

## Figures and Tables

**Table 1:** Recombination information.

Event No.	Found In	Recombinant	Major Parent	Minor Parent	Detection Methods						
					R	G	B	M	C	S	T
1	1	OP233011	FJ644932	Unknown	−	−	−	+	+	+	+
2	2	JX099786	ON012608	Unknown	−	−	−	+	−	+	+
3	4	EF421973	DQ180393	HM776452	−	−	−	+	−	−	+
4	2	HQ591381	JX506730	EU148503	−	−	−	+	+	−	−
5	4	MH656967	OP233015	Unknown	−	−	−	+	−	−	−
6	3	JQ181592	EU148503	ON012608	−	−	−	+	−	+	+
7	20	HM776452	AF465211	EU148505	+	+	−	−	−	−	+
8	3	EU148505	KJ187306	Unknown	−	−	−	−	+	−	−
9	3	EU148504	Unknown	JQ955679	−	−	−	−	−	+	−

Note: RDP (R), GENECONV (G), Bootscan (B), Maxchi (M), Chimaera (C), SiSscan (S), and 3Seq (T). “+” means possible recombination, and “−” means no recombination.

**Table 2:** Mutations in the Cap and Rep proteins of PCV2 isolates compared with that of the AY391729 (PCV2b), AY291317 (PCV2d), and GU001709 (PCV2e).

Protein	Reference Strain	Isolates	Genotype	Mutation Site
Cap	AY391729	ON012605	2b	R59K, K63R, K180R, F221S
		ON012608	2b	L19P, R59K, K63R, K180R
		OP233009, OP233013, OP233014, OP279265, OP279266, OP279267, OP279268	2b	R59K, K63R, K180R
Cap	AY291317	OP233011	2d	Y8F, A59K, P151T, M217L
		OP233012, OP279264	2d	Y8F, A59K, P151T, R169G, N178S
Cap	GU001709	OP233015, OP233008, OP233010	2e	V123I
		OP233007, ON012607	2e	T60S, V123I
		ON012606	2e	None
Rep	AY391729	OP279266	2b	D34E, V65M, L77F
		OP279268, ON012605, ON012608, OP233009, OP233013, OP233014, OP279265, OP279267	2b	D34E, L77F

Note: consensus mutations are underlined.

**Table 3:** Effect of mutations on the secondary structure of the Cap and Rep proteins.

		Clash	Buried Hydrophilic Introduced	Buried Charge Introduced	Secondary StructureAltered	Buried Charge Switch	Disallowed phi/psi	Buried Charge Replaced	Buried H–Bond Breakage	Cavity Altered	Buried/Exposed Switch
AY391729 rep	D34E	+−−	+++	+++	NA	+++	++−	+++	+++	−−−	+++
AY391729 rep	V65M	+−−	+++	+++	NA	+++	+−−	+++	−−−	−−−	+++
AY391729 rep	L77F	+++	+−−	+−−	NA	+−−	++−	+−−	−−−	−−−	+−−
AY391729 Cap	R59K	−−−	+−−	+−−	NA	+−−	++−	+−−	+++	−−−	+−−
AY391729 Cap	K63R	−−−	+++	+++	NA	+++	++−	+++	+++	−−−	+++
AY391729 Cap	K180R	+−−	+++	+++	NA	+++	++−	+++	+++	+++	+++
AY391729 Cap	F221S	+−−	+−−	+−−	NA	+−−	++−	+−−	−−−	−−−	+−−
AY391729 Cap	L19P	+++	++−	++−	NA	++−	+−−	++−	−−−	++−	++−
AY291317 Cap	Y8F	+−−	++−	++−	NA	++−	++−	++−	+++	+++	++−
AY291317 Cap	A59K	−−−	+++	+++	NA	+++	++−	+++	+++	+−−	+++
AY291317 Cap	P151T	+−−	++−	++−	NA	++−	++−	++−	−−−	−−−	++−
AY291317 Cap	M217L	++−	+−−	+−−	NA	+−−	++−	+−−	−−−	+++	+−−
AY291317 Cap	R169G	+−−	+++	+++	NA	+++	++−	+++	+++	−−−	+++
AY291317 Cap	N178S	++−	+++	+++	NA	+++	++−	+++	+++	+++	+++
GU001709 Cap	V123I	+−−	+−−	+−−	NA	+−−	++−	+−−	+−−	++−	+−−
GU001709 Cap	T60S	+−−	+−−	+−−	NA	+−−	++−	+−−	+++	+−−	+−−

Note: “+” means difference, and “−” means no difference. The number of “+” or “−” represents the quantity of different or similar, respectively. NA, none.

## Data Availability

All data generated or analyzed during this study are included in this published article and its Supplementary file.

## Supplementary Materials

The following supporting information can be downloaded at: https://www.mdpi.com/article/10.3390/microorganisms11040983/s1, Table S1: Information of PCV2 isolates in this study; Table S2: The complete genome sequences of PCV2 reference strains; Table S3: Mutations in B cell epitopes of Rep proteins of PCV2 isolates compared with three commercial reference vaccine strain; Table S4: Mutations in B cell epitopes of Cap proteins of PCV2 isolates compared with three commercial reference vaccine strain; Table S5: Mutations in T cell epitopes of Cap proteins of PCV2 isolates compared with three commercial reference vaccine strain; Supplementary Figure S1: Predicated 3D structures of PCV2 Cap and Rep proteins from different genotypes.
