# Supplementary material for: Phylogenetic and Structural Analysis of Porcine Circovirus Type 2 from 2016 to 2021 in Jilin Province, China
Source: Microorganisms. 2023 Apr 10;11(4):983. doi: 10.3390/microorganisms11040983 (PMC10145682; doi:10.3390/microorganisms11040983)
Supplement: Supplementary file 1 [file microorganisms-11-00983-s001.zip › microorganisms-2285139-supplementary.pdf]

Supplementary Table S1 Information of PCV2 isolates in this study.

Accession number	Length	Genotype	Year of collection	Tissue	Major symptoms
ON012605	1767	2b	2017	lymph node	Fever, diarrhea
ON012608	1767	2b	2020	lymph node	Fever, anorexia
OP233009	1767	2b	2016	lymph node	Pale skin, dyspnea
OP233013	1767	2b	2019	lymph node	Dyspnea, cough
OP233014	1767	2b	2020	lymph node	Fever, anorexia
OP279265	1767	2b	2021	lymph node	anorexia
OP279266	1767	2b	2021	tonsil	Fever
OP279267	1767	2b	2018	tonsil	Dyspnea, anorexia
OP279268	1767	2b	2016	tonsil	Fever, cough
OP233011	1767	2d	2019	tonsil	Dyspnea, anorexia, diarrhea
OP233012	1767	2d	2021	tonsil	Fever, neurological symptoms
OP279264	1767	2d	2021	lymph node	Cough, anorexia
ON012606	1768	2e	2018	lymph node	Fever, leg weakness
ON012607	1768	2e	2020	lymph node	Fever, anorexia

OP233007	1768	2e	2020	lymph node	Pale skin, dyspnea
OP233008	1768	2e	2017	tonsil	Fever, dyspnea
OP233010	1768	2e	2016	tonsil	Cough, diarrhea, anorexia
OP233015	1768	2e	2019	tonsil	Fever, anorexia

Supplementary Table S2 The complete genome sequences of PCV2 reference strains.

Accession number	Strains	Origin	Length	Genotype	Release Date
KJ187306	PCV2-UFV1	Brazil	1767	2d	2014
AF465211	SC	China	1768	2a	2002
AY291317	HB	China	1767	2d	2003
AY391729	NB0301	China	1767	2b	2003
AY556473	SD	China	1767	2d	2004
DQ104423	DTC	China	1768	2a	2005
DQ180393	putian0401	China	1767	2b	2005
EF421973	Huanan-8	China	1767	2b	2007
EF524539	TJ06	China	1767	2d	2007
EF524532	GX0601	China	1768	2e	2007
EF524537	HB0602	China	1768	2e	2007
FJ644930	HM08	China	1767	2b	2009
FJ644932	SF08	China	1767	2b	2009
GU001710	BJ0901b	China	1767	2d	2009
GU001709	BJ0901a	China	1768	2e	2009
HM776452	YN-8	China	1767	2f	2011
JQ955679	CC1	China	1766	2b	2012
MH656967	YN-LJ-2018	China	1768	2a	2018
MK424114	YN/LiJiang-2017	China	1768	2a	2019
HQ591381	1314-09-1	Croatia	1767	2f	2011

EU148503	DK1980PMWSfree	Denmark	1767	2c	2007
EU148504	DK1987PMWSfree	Denmark	1767	2c	2007
EU148505	DK1990PMWSfree	Denmark	1767	2c	2007
DQ233257	ROM	Hungary	1767	2b	2005
KP231168	961_Padova_11_13/01/2014	Italy	1767	2d	2015
KP420197	ZrBd wb UKR	Ukraine	1767	2g	2017
JQ181592	BG0-1	Viet Nam	1767	2h	2012
JX099786	P2425NT	Viet Nam	1767	2g	2013
JX506730	NAVET_vietnam3	Viet Nam	1767	2h	2012
KM042398	549-QNa	Viet Nam	1767	2h	2016

Supplementary Table S3 Mutations in B cell epitopes of Rep proteins of PCV2 isolates compared with three commercial reference vaccine strain.

Reference strain	B Cell Epitopes	Isolates			
		OP279266	ON012606	OP233012, OP279264	ON012605, ON012607, ON012608, OP233010OP233011, OP233013, OP233014, OP233015, OP279265, OP279267, OP279268, OP233007, OP233008, OP233009
AY686763	S^{3}KKNGRSGPQ^{12}			**N6S**	
	H^{57}LQGFANFVK^{66}	**V65M**			
	P^{110}RSQGQRSDL^{119}		**S112C**		
	N^{168}EHVIVGPPG^{177}	**E169V**	**E169V**	**E169V**	**E169V**
HM641752	S^{3}KKNGRSGPQ^{12}			**N6S**	
	S^{25}EDERKKIRD^{34}	**D34E**	**D34E**	**D34E**	**D34E**
	H^{57}LQGFANFVK^{66}	**V65M**			
	P^{110}RSQGQRSDL^{119}		**S112C**		
HM038034	S^{3}KKNGRSGPQ^{12}			**N6S**	
	H^{57}LQGFANFVK^{66}	**V65M**			
	P^{110}RSQGQRSDL^{119}		**S112C**		

Supplementary Table S4 Mutations in B cell epitopes of Cap proteins of PCV2 isolates compared with three commercial reference vaccine strain.

Reference strain	B Cell Epitopes	Isolates				
		OP233011, OP233012, OP279264	ON012606	OP233015, ON012607, OP233007, OP233008, OP233010	ON012608	ON012608, OP233009, OP279267, OP279268, OP279265, OP279266, OP233013, OP233014, ON012605
AY686763	T^{2}YPRRRYRRR^{11}	**Y8F**				
	R^{7}YRRRRHRPR^{16}	**Y8F**				
	S^{17}HLGQILRRR^{26}				**L19P**	
	Q^{21}ILRRRPWLV^{30}				**L21Q**	
	I^{44}FNTRLSRTI^{53}		**T47S, I53F**	**T47S, I53F**		**I53F**

	V^{57}KATTVRTPS66^{66}					**V57I**
	P^{65}SWAVDMMRF^{74}	**A68N**				
	T^{121}AVILDDNFV^{130}		**T121S**	**T121S, V123I**		**T121S**
HM641752	T^{2}YPRRRYRRR^{11}	**Y8F**				
	R^{7}YRRRRHRPR^{16}	**Y8F**				
	S^{17}HLGQILRRR^{26}				**L19P**	
	I^{44}FNTRLSRTF^{53}		**T47S**	**T47S**		
	I^{57}KRTTVKTPS^{66}	**I57V**	**I57V**	**I57V**		
	P^{65}SWAVDMMRF^{74}	**A68N**				
	S^{121}AVILDDNFV^{130}	**S121T**	**V130F**	**V123I, V130F**		
HM038034	R^{5}RRFRRRRHR^{14}		**F8Y**	**F8Y**		**F8Y**
	S^{17}HLGLILRRR^{26}				**L19P**	
	L^{21}ILRRRPWLV^{30}	**L21Q**	**L21Q**	**L21Q**	**L21Q**	**L21Q**
	I^{44}FNTRLSCTF^{53}		**T47S**	**T47S**		
	V^{57}KATTVRTPS^{66}					**V57I**
	P^{65}SWAVDMMRF^{74}	**A68N**				
	N^{87}EISIPFEYY^{96}	**E88P, I89L, S90T, I91V**	**E88K**	**E88K**		**E88P, I89R, I91V**

Supplementary Table S5 Mutations in T cell epitopes of Cap proteins of PCV2 isolates compared with three commercial reference vaccine strain.

Reference strain	T Cell Epitopes	Isolates		
		**OP233012, OP279264, OP233011**	**OP233015, OP233007, OP233008, ON012606, ON012607, OP233010**	**OP233009, OP279267, OP279268, OP279265, OP279266, OP233013, OP233014, ON012605, ON012608**
AY686763	V^{130}TKANALTY^{138}		**V130F, T131P, A133S, N134T**	**N134T**
	H^{148}TIPQPFSY^{156}	**P151T**		**P151T**
	G^{199}TAFENSKY^{207}	**K206I**		**K206I**
HM641752	V^{130}TKATALTY^{138}	**T134N**	**V130F, T131P, A133S**	

	A^{133}TALTYDPY^{141}		**A133S**	
	H^{148}TITQPFSY^{156}	**T151P**	**T151P**	
	G^{199}TAFENSIY^{207}		**I206K**	
HM038034	V^{130}TRATALTY^{138}	**T134N R132K**	**V130F, T131P, R132K, A133S**	**R132K**
	A^{133}TALTYGPY^{141}	**G139D**	**G139D**	**G139D**
	H^{148}TIPQPFSY^{156}	**P151T**		**P151T**
	G^{199}IAFENSTY^{207}	**T206I**	**T206K**	**T206I**

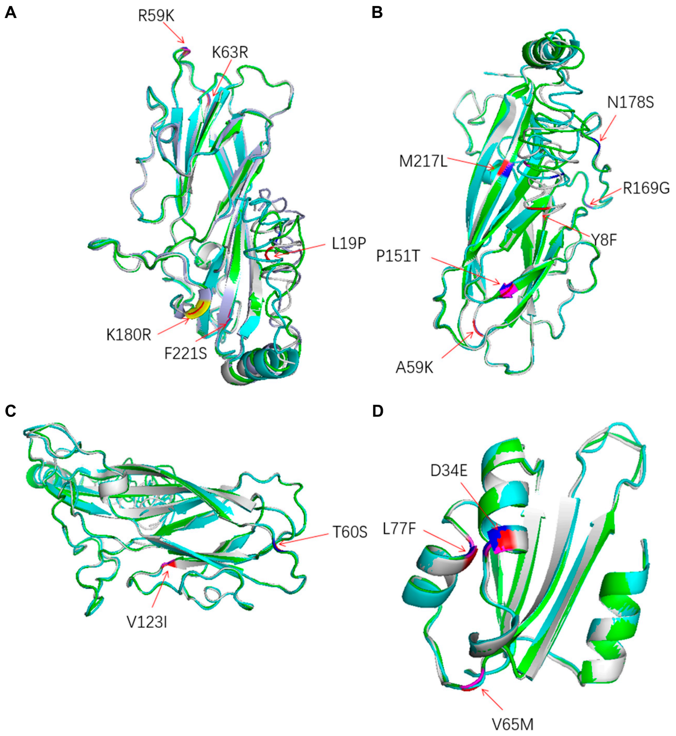

Supplementary Figure S1 Predicated 3D structures of PCV2 Cap and Rep proteins from different genotypes. (A) PCV2b Cap; (B) PCV2d Cap; (C) PCV2e Cap; (D) PCV2b Rep proteins. Isolates AY391729, AY291317, and GU001709 were reference sequences marked as white. Isolates ON012605, OP233011, OP233015, and OP279266 were labeled in green. Isolates ON012608, OP233012, OP233007, and OP279268 were tagged with cyan. Isolate OP233009 was marked by wheat. Different amino acids of reference sequences were highlighted in red, and that of ON012605, OP233011, OP233015, and OP279266 were highlighted in magenta. Mutated amino acids of ON012608, OP233012, OP233007, and OP279268 were highlighted in blue, and that of OP233009 was highlighted in yellow.
